# Early hippocampal high-amplitude rhythmic spikes predict post-traumatic epilepsy in mice

**DOI:** 10.3389/fnins.2024.1422449

**Published:** 2024-08-29

**Authors:** Tyler Shannon, Noah Levine, Rina Dirickson, Yuyan Shen, Christopher Cotter, Noora Rajjoub, Julie Fitzgerald, Fernando Pardo-Manuel de Villena, Olga Kokiko-Cochran, Bin Gu

**Affiliations:** ^1^Department of Neuroscience, Ohio State University, Columbus, OH, United States; ^2^Electrical and Computer Engineering Program, Ohio State University, Columbus, OH, United States; ^3^College of Veterinary Medicine, Ohio State University, Columbus, OH, United States; ^4^Institute for Behavioral Medicine Research, Ohio State University, Columbus, OH, United States; ^5^Department of Genetics, University of North Carolina, Chapel Hill, NC, United States; ^6^Lineberger Comprehensive Cancer Center, University of North Carolina, Chapel Hill, NC, United States; ^7^Chronic Brain Injury Program, Ohio State University, Columbus, OH, United States

**Keywords:** brain oscillation, traumatic brain injury, epilepsy, post traumatic epilepsy, biomarkers, EEG, local field potential (LFP), collaborative cross mice

## Abstract

Oscillations, a highly conserved brain function across mammalian species, play a pivotal role in both brain physiology and pathology. Traumatic brain injury (TBI) frequently results in subacute and chronic alterations in brain oscillations, which are often associated with complications like post-traumatic epilepsy (PTE) in patients and animal models. We recently conducted longitudinal recordings of local field potential from the contralateral hippocampus of 12 strains of recombinant inbred Collaborative Cross (CC) mice and classical laboratory inbred C57BL/6 J mice after lateral fluid percussion injury. In this study, we profiled the acute (<12 h post-injury) and subacute (12–48 h post-injury) hippocampal oscillatory responses to TBI and evaluated their predictive value for PTE. We found dynamic high-amplitude rhythmic spikes with elevated power density and reduced signal complexity that prevailed exclusively during the acute phase in CC031 mice, which later developed PTE. This characteristic early brain oscillatory alteration was absent in CC031 sham controls, as well as in other CC strains and reference C57BL/6 J mice that did not develop PTE after TBI. Our findings offer quantitative measures linking early hippocampal brain oscillation to PTE at a population level in mice. These insights enhance understanding of circuit mechanisms and suggest potential targets for neuromodulatory intervention.

## Introduction

Traumatic brain injury (TBI) results in a range of neurological conditions, including sleep disturbances, cognitive impairments, psychiatric complications, and recurrent seizures. Among them, post-traumatic epilepsy (PTE) emerges as one of the most prevalent and debilitating post-injury disorders. PTE significantly impacts individuals’ quality of life and is associated with heightened risks of unfavorable functional outcomes and mortality (Englander et al., 2009). The increased susceptibility of epilepsy and its related comorbidities following TBI underscores the critical need for novel resources and tools to identify individuals at risk for PTE. Despite extensive research aimed at identifying biomarkers for TBI outcomes and epilepsy, there remains a paucity of studies investigating specific biomarkers for post-traumatic epileptogenesis.

Electroencephalography (EEG) is a widely adopted noninvasive neurodiagnostic tool for examining brain oscillatory function in TBI and epilepsy. However, the interpretation of EEG findings post-TBI remains challenging due to their heterogeneous nature, with early studies yielding conflicting results regarding its predictive utility for PTE (Perucca et al., 2019). A collection of descriptive and quantitative EEG alterations have been documented following TBI, including early post-traumatic seizures, high amplitude sharp waves, high-frequency oscillation (HFO), repetitive HFOs and spikes, sleep spindle duration, and bidirectional changes in EEG power (Haneef et al., 2013; Perucca et al., 2019). Retrospective case-controlled studies suggest some of these quantitative EEG features hold promise as predictive indicators for PTE (Bragin et al., 2016; Reid et al., 2016; Chen et al., 2023; Pease et al., 2023). However, these patient studies face limitations such as poorly controlled environments, selection bias toward severe injury cases, and insufficient consideration of patients’ genetics, demographic information, comorbid conditions, or medication history (Rubinos et al., 2022). Though animal models offer greater control over environmental variability, early animal studies of TBI commonly use a single laboratory inbred strain, failing to capture the genetic diversity seen in humans (Brady et al., 2019; Hajiaghamemar et al., 2019). For example, the genotype–phenotype relationships observed in a single genetic background may not be generalizable to others, limiting the translational relevance of findings derived from such models (Sittig et al., 2016).

We recently conducted a comprehensive characterization of TBI-related outcomes across 12 strains of the Collaborative Cross mice (CC), a next-generation multi-parental recombinant inbred mouse panel, in conjunction with the commonly used classical inbred C57BL/6 J (B6J) mice (Shannon et al., 2024). The CC provides a population of genetically diverse mice that enables systematic studies of acute, subacute, and chronic brain oscillatory responses to TBI and how this information relates to PTE in a controlled environment. Among the 13 mice strains studied, CC031 emerged as the sole mouse strain displaying frequent spontaneous seizures after a moderate brain injury induced by lateral fluid percussion injury (FPI) (Shannon et al., 2024). Leveraging this novel dataset, we quantitatively analyzed acute (< 12 h) and subacute (12–48 h) post-traumatic oscillatory features in epileptic CC031 mice (CC031-TBI), comparing them to non-epileptic CC and B6J strains that do not develop PTE after the same injury (Supplementary Figure 1). We further analyzed new data collected from sham-operated CC031 and B6J mice as critical controls for injury-dependent phenotypes. This expanded dataset allows us to delineate temporal brain oscillatory features associated with PTE in consideration of genetic and injury dependences. A deeper understanding of the dynamic changes in brain oscillation post-TBI holds promise for identifying biomarkers and developing therapeutic interventions for PTE.

## Methods

### Animals

All CC strains were acquired from the Systems Genetics Core Facility at the University of North Carolina (UNC) at Chapel Hill between 2021 and 2023, including CC008/GeniUnc, CC012/GeniUnc, CC013/GeniUnc, CC021/Unc, CC025/GeniUnc, CC027/GeniUnc, CC031/GeniUnc, CC038/GeniUnc, CC044/Unc, CC053/Unc, CC058/Unc, and CC071/TauUnc (referred herein as CC008, CC012, CC013, CC021, CC025, CC027, CC031, CC038, CC044, CC053, CC058, and CC071, respectively). B6J mice (#000664) were purchased from the Jackson Laboratory. Adult (3–6 months) male mice were used in this study. For TBI group, we used CC008 (*n* = 3), CC012 (*n* = 6), CC013 (*n* = 4), CC021 (*n* = 8), CC025 (*n* = 4), CC027 (*n* = 3), CC031 (*n* = 9), CC038 (*n* = 6), CC044 (*n* = 6), CC053 (*n* = 7), CC058 (*n* = 4), CC071 (*n* = 6), and B6J (*n* = 10) mice across 11 cohorts. High mortality after TBI was reported in CC027 (Shannon et al., 2024), which were excluded from the analysis. For the sham-operated control group, we included CC031 (*n* = 7) and B6J (*n* = 5) mice over three cohorts. Various sample sizes across strains were the results of pooled data from an original tiered phenotypic screening (Shannon et al., 2024) and extended characterization of traits of interest (e.g., PTE). All procedures were performed under the Institutional Animal Care and Use Committee (IACUC) of Ohio State University.

### Surgery and lateral FPI

Mice were anesthetized using isoflurane. Following a midline incision, a 3 mm diameter craniectomy was performed over the right hemisphere (coordinates from bregma: −0.5 mm AP and −2.5 mm ML) with careful attention to leaving the dura matter intact. A modified portion of a Luer-Lock (3 mm inner diameter) was positioned over the exposed dura and then secured over the craniectomy by cyanoacrylate adhesive and dental acrylic. Mice were returned to the home cage to recover overnight. One day after craniectomy, lateral FPI was delivered in isoflurane-anesthetized mice. Diffuse TBI was induced by delivering a 10–20 ms pulse of saline (1.2–1.5 atmospheres) onto the intact dura through the injury hub, which is equivalent to moderate TBI, using an FPI apparatus (Custom Design and Fabrication, Richmond, VA). A separate group of age-matched CC031 and B6J mice was subject to sham operation and recorded as controls. Animals in the sham group were connected to the injury device for the same period of time; however, no fluid pulse was delivered. After FPI or sham injury, the Luer-Lock and adhesive were removed. The self-righting reflex test was recorded using a timer as a surrogate measurement of injury severity. A stainless steel bipolar recording electrode (Protech International Inc.) was then stereotaxically placed into the left dorsal hippocampus (coordinates from bregma: −2.0 mm AP, 1.6 mm ML, and 1.0 mm below dura). A ground electrode was fastened to a stainless steel screw on the skull above the left olfactory bulb. The electrodes were fixed, and the opening was closed using dental acrylic.

### Video-local field potential (LFP) monitoring

We performed time-locked video-LFP recordings across three time frames following injury: week 1, week 6, and week 9–10 post-injury. This design allows us to study early brain oscillatory biomarkers that can shape the later onset of PTE. However, the absence of seizure within 10 weeks post-injury does not exclude the possibility that seizures may emerge later, beyond the end of our recording window. Longer recordings are required to ensure the absence of PTE. During recordings, the mouse was single-housed under diurnal conditions of 12/12 light and dark cycles. The recordings were started ~3 h after completion of TBI to allow the animal to recover and regain voluntary activity from injury and surgery. The 24/7 continuous recordings were performed using a 12-channel acquisition system (Data Science International). Ponemah 5.5 software was used for data acquisition and video synchronization (infrared-enabled for night vision). The mouse can freely roam within the housing cage via a tethered system connected to a commutator. LFP recordings were sampled at a rate of 2,000 Hz.

### Open field tests

On 10 days post-injury (during the break between week 1 and week 6 post-injury recording sessions). Each mouse was placed in a polypropylene open-field arena (36 cm × 36 cm) with two rows of infrared sensors mounted on the sides to detect and distinguish between horizontal and vertical movements during a 10-min session. Activity counts were defined as interruptions in the infrared light sources by the animal (i.e., beam breaks). Distance traveled and the number of rears were analyzed.

### Signal processing and analysis

All LFP data were filtered using an eighth-order notch filter of 60 Hz before analysis. The power spectrum and phase-amplitude coupling were analyzed using *Brainstorm* and customized MATLAB algorithms. The spectrogram, phase preference, and entropy were analyzed and visualized using MNE and Tensorpac packages in Python. A random 2-min time window was selected during specific time frames of 3–12 h (acute) and 12–48 h (subacute) after TBI. The length of the time window was selected to reflect the patterns and features of LFP while considering the computational cost. For the power spectrum, the LFP data were firstly transformed using Morlet Wavelet with 3 s time resolution and 0.5 Hz step size. The data were further divided into particular wave bands for further quantification: delta (0.5–4 Hz), theta (4–8 Hz), alpha (8–12 Hz), and beta (12–30 Hz). The performance of the PTE prediction model was evaluated using the receiver operating characteristic (ROC) and the area under the curve (AUC) ROC analysis. The spectrogram scale was adjusted to reveal details of the sample with the lowest power. For phase-amplitude coupling (PAC), the time-resolved PAC was computed using a 4-s sliding time window. We generated the average comodulogram in the frequency ranges for phase (fP, 0.5–15 Hz) and frequency for amplitude (fA, 30–200 Hz). The coupling strength of delta/low gamma with fP (0.5–4 Hz)/fA (30–70 Hz) and delta/high gamma with fP (0.5–4 Hz)/fA (80–200 Hz) was calculated. For phase preference, we computed target fP of 0.5–4 Hz, minimum fA of 30 Hz, and maximum fA of 100 Hz with a 4-s sliding time window using Tensorpac in Python. The average phase preference was computed and plotted in a polar histogram. Entropy was calculated using the app_entropy, samp_entropy, and spect_entropy functions of MNE in Python.

### Immunohistochemistry and imaging analysis

The mouse was euthanized after the completion of the recordings. The brain tissue of each mouse was fixed in 4% paraformaldehyde and cryoprotected using 30% sucrose. Serial 40 μm coronal sections were sliced through the forebrain, spanning the entire dorsal hippocampal area. Sections were incubated with anti-Iba1 (1:1,000, FUJIFILM Wako 019-19741) followed by Alexa-555 (1:1,000, Invitrogen A21428). Images were acquired on an Olympus FV3000 spectral confocal microscope using a 10x/0.3 objective. For each animal, three adjacent slices were imaged and averaged per mouse as one biological replicate. Using ImageJ, the images were first thresholded and the immunoreactivity in regions of interest (ROIs) above the threshold was measured. The percent area of Iba1 immunoreactivity was measured in an ROI (730 μm × 730 μm) contoured around the medial cortex (MC), CA1, and CA3. Colabeling of DAPI (1:10,000, Life Technologies) was used as an indicator of the cell layers.

### Statistical analysis

All experiments and analyses were performed by investigators who were blind to mouse strains and experimental groups. The Shapiro–Wilk normality test was performed to justify parametric statistical tests assuming Gaussian distribution. If data failed the normality test, we applied the appropriate non-parametric statistical tests instead. All data were presented as mean ± SEM. Unless otherwise noted, comparisons were analyzed using paired Student’s *T*-test or two-way ANOVA with Šídák’s *post hoc* test. *p* < 0.05 was considered statistically significant.

## Results

### CC031 mice exhibited injury-dependent post-traumatic seizures

Our recent work found CC031 mice, but not the rest of the 11 strains of CC or B6J, exhibited high incident and frequent spontaneous seizures (4.1 PTS/week, week 9–10 post-injury in eight out of nine mice) after a moderate brain injury induced by lateral FPI (Shannon et al., 2024). The PTS in CC031 are characterized by episodes of electrographic seizures with clear initiation and termination, accompanied by behavioral manifestations such as whole-body colonus (Shannon et al., 2024) (Supplementary Video 1). To further confirm the association between the PTS and TBI in CC031 mice and compare their quantitative EEG features in the presence and absence of injury, we challenged a separate cohort of B6J and CC031 mice with sham operation. We found TBI significantly increased righting reflex time immediately following injury (Supplementary Figure 2) and Iba1 immunoreactivity 70 days post-injury (Supplementary Figure 3), regardless of genotype, confirming the extent of the injury. Notably, none of the CC031-sham mice exhibited PTS during the same post-operative recording windows. This observation suggests that the PTE phenotype of CC031 is injury-dependent, indicating that the genetic predisposition of CC031 mice renders them more susceptible to PTE following brain injury.

### Dynamic high-amplitude rhythmic spikes prevail in the contralateral hippocampus during the acute phase after TBI in mice with PTE

**Figure 1:**
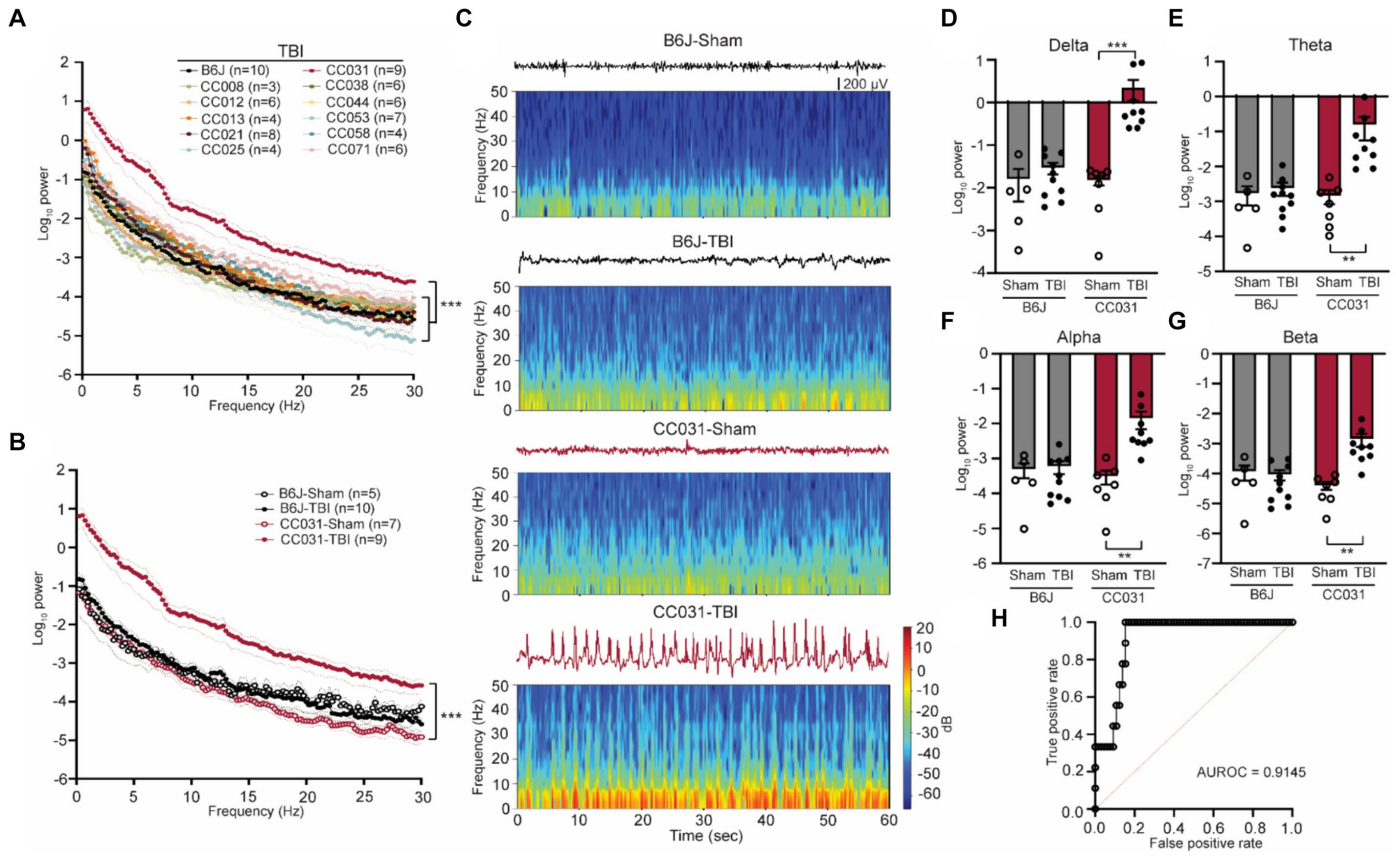
Acute contralateral hippocampal DHRS precedes and predicts PTE. **(A)** Power spectral density analysis across 11 CC strains and B6J mice within 12 h after TBI. Data are presented as mean ± SEM and analyzed using two-way ANOVA with *post hoc* Dunnett’s multiple comparison test, *n* = 3–10, ^***^p < 0.001. **(B)** Power spectral density analysis of CC031 and B6J mice within 12 h after TBI (replot) and their sham controls. Data are presented as mean ± SEM and analyzed using two-way ANOVA, ^***^p < 0.001, *n* = 5–10. **(C)** Representative EEG and spectrogram of CC031 and B6J mice within 12 h after sham operation and TBI. **(D–G)** Accumulated power in **(D)** delta, **(E)** theta, **(F)** alpha, and **(G)** beta frequency bands in B6J and CC031 mice who were subject to sham operation or TBI. Data are presented as mean ± SEM and analyzed using two-way ANOVA with Šídák’s multiple comparisons test. *n* = 5–10, ^**^p < 0.01 and ^***^p < 0.001. **(H)** ROC curve of logistic regression of logarithm of delta power in predicting the occurrence of PTE (*n* = 76). AUROC = 0.9145, 95% CI: 0.8474–0.9817.

**Figure 2:**
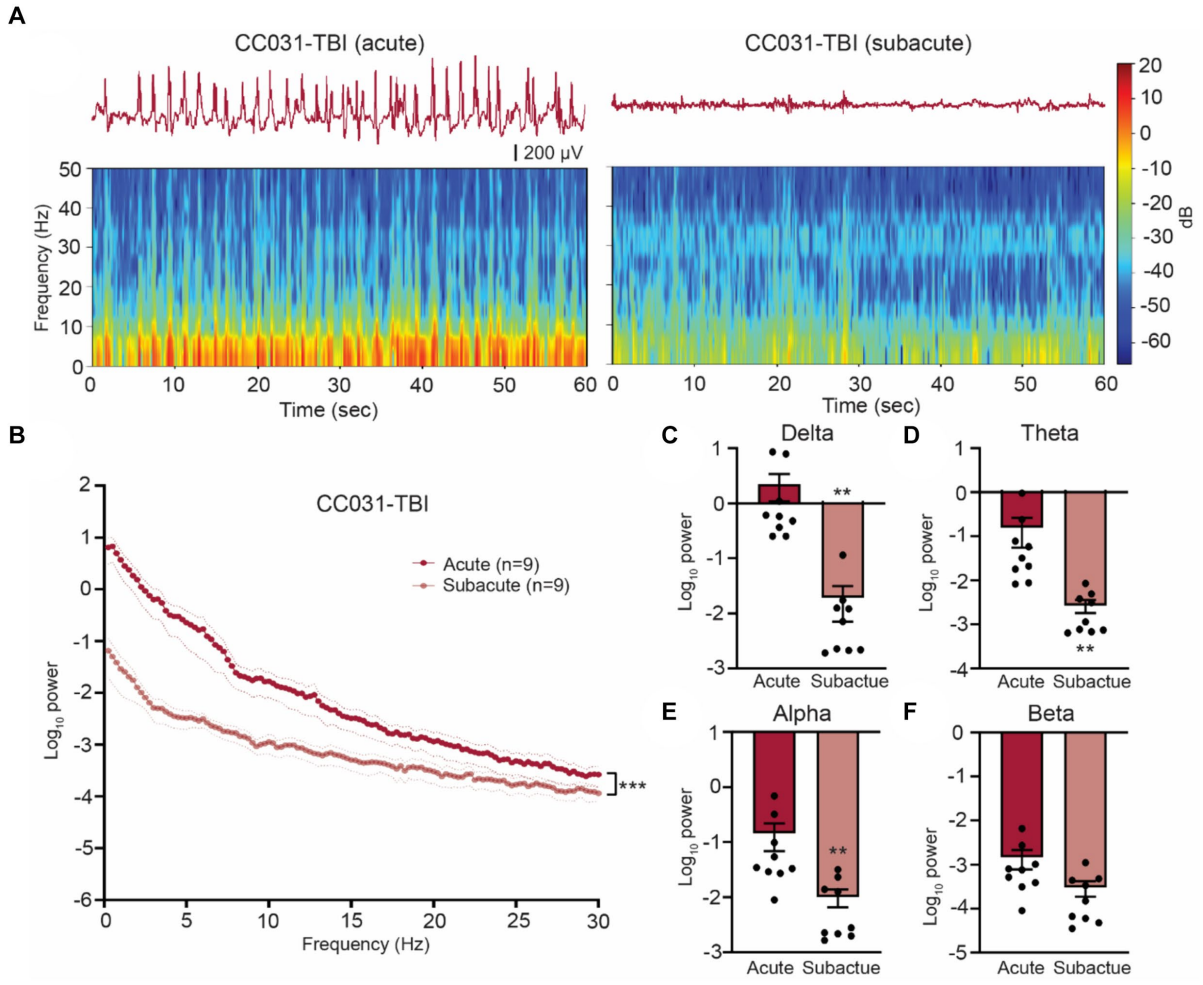
Hippocampal LFP resumes normality after acute phase post injury in CC031 mice. **(A)** Representative LFP traces and spectrograms during acute and subacute phases in a CC031-TBI mouse. **(B)** Power spectral density analysis of CC031-TBI mice during acute vs. subacute phases. Data are presented as mean ± SEM and analyzed using two-way ANOVA, ^***^p < 0.001. **(C–F)** Accumulated power in **(C)** delta, **(D)** theta, **(E)** alpha, and **(F)** beta frequency bands of CC031-TBI mice comparing acute vs. subacute phases. Data are presented as mean ± SEM and analyzed using Wilcoxon matched-pairs signed rank test, ^**^p < 0.01.

Unlike most previous preclinical EEG studies in TBI, which focused on analyzing the EEG recordings collected days, weeks, and months after injury, our study specifically focused on the acute (<12 h) and subacute (12–48 h) time windows after TBI. This early post-injury phase is crucial yet often understudied, as the brain oscillatory alterations during this phase may reflect the immediate responses like blood–brain barrier breakdown (Seiffert et al., 2004; Tomkins et al., 2011) and hypermetabolism (Koenig and Dulla, 2018) that play a critical role in the pathogenesis of PTE. Through manual inspection of the initial 48 h of LFP recordings covering both acute and subacute phases after TBI, we identified distinct electrographic abnormalities characterized by 0.3–0.5 Hz high voltage rhythmic spikes, referred to as dynamic high-amplitude rhythmic spikes (DHRS), exclusively during the acute phase in all CC031-TBI mice (Supplementary Figures 4, 5). Importantly, DHRS was absent in non-epileptic mice strains subjected to TBI or sham-operated B6J and CC031 controls (Figures 1A–C). We also found these DHRS were temporally restricted, occurring exclusively during the acute phase (average latency to onset = 386 ± 64 min and average duration = 178 ± 50 min), with the brain oscillations resuming normality afterward until the emergence of PTE (Figure 2). Notably, DHRS was also observed in one CC031 mouse who did not exhibit PTS during the three chronic recording sessions after TBI. However, longer continuous recordings are required to prove the presence or absence of seizures in this particular mouse. In sum, our findings demonstrate the presence of DHRS with characteristic power features exclusively in the mouse strain predisposed to PTE, highlighting a refined time window immediately following brain trauma for assessing PTE risks.

### The power spectral density of DHRS predicts PTE

To further quantitatively analyze the energy of DHRS, we computed power spectral density using a randomly selected artifact-free two-minute time window during each phase, ensuring adequate capture of DHRS electrographic characteristics. During the acute phase, CC031-TBI mice exhibited a significant ubiquitous power elevation across the 0–30 Hz frequency domains compared to non-epileptic mouse strains (Figure 1A). Given the comparable LFP features between B6J and the CC strains that did not develop PTE and the impetus to use classical inbred mice, subsequent analyses focused on comparing epileptic CC031-TBI mice with non-epileptic control groups, including CC031-sham, B6J-sham, and B6J-TBI. We found the power density increased across the delta, theta, alpha, and beta bands in CC031-TBI mice compared to non-epileptic control groups (Figures 1B–G). Logistic regression analysis of the logarithm of delta power revealed promising predictive utility for PTE, with an AUROC value of 0.9145 (Figure 1H). These findings underscore the potential of quantitative brain oscillatory features, particularly acute delta power, in discriminating PTE and hold implications for predictive modeling.

### Quantitative brain oscillatory features delineate PTE

**Figure 3:**
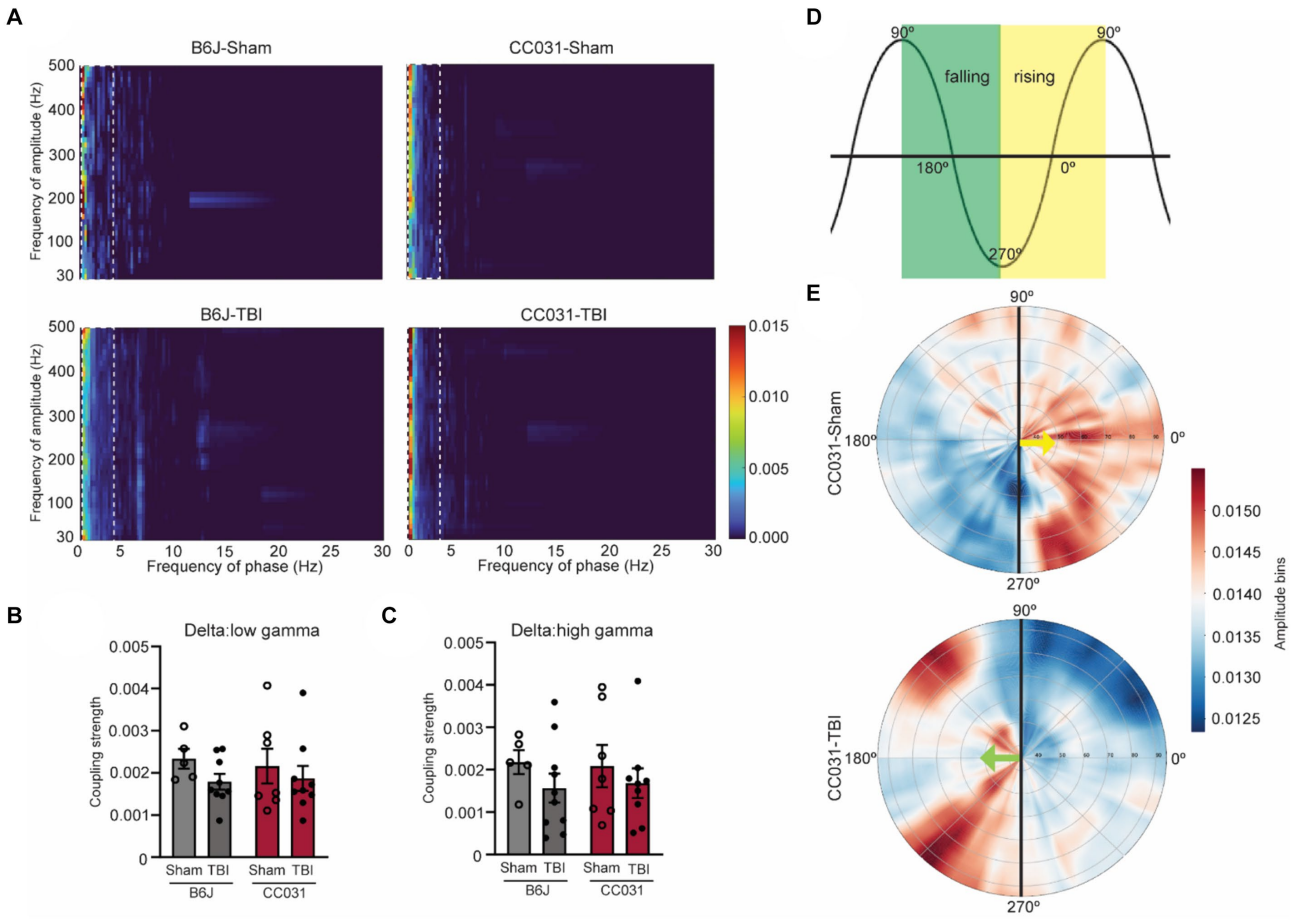
PAC and phase preference analyses in the hippocampus of mice. **(A)** Average comodulogram of hippocampal PAC (fP: 0.5–30 Hz/fA: 30–500 Hz) in B6J-Sham, B6J-TBI, CC031-Sham, and CC031-TBI mice. The dashed rectangle denotes the dominant delta/gamma PAC (fP: 0.5–4 Hz/fA: 30–500 Hz). **(B,C)** Average coupling strength of delta/low gamma (fP: 0.5–4 Hz/fA: 30–70 Hz) and delta/high gamma (fP: 0.5–4 Hz/fA: 80–200 Hz, top row) PAC ranges. Data are presented as mean ± SEM and analyzed using two-way ANOVA with Šídák’s multiple comparisons test, *n* = 5–10. **(D)** Schematic of phase angle. The green square denotes the falling phase. The yellow square indicates the rising phase. **(E)** The angle represents the delta frequency (0.5–4 Hz) phase, and the radial axis represents gamma frequencies (30–100 Hz) for the amplitude signal. The color depicts the average amplitude value of a given frequency inside the corresponding phase bin. Gamma oscillation (30–100 Hz) preferably riding on the rising phase of the delta wave in CC031-Sham mice while on the falling phase of the delta wave in CC031-TBI mice.

In addition to power analysis, we computed time-resolved PAC, a physiologically relevant measure prominent in the hippocampus that links to cognition and epilepsy (Antonakakis et al., 2016; Liu et al., 2017). We found that cross-frequency coupling of delta and gamma oscillations (fP: 0.5–4 Hz/fA: 30–200 Hz) was dominant in all four groups of CC031-Sham, CC031-TBI, B6J-sham, and B6J-TBI mice (Figure 3A). Subsequently, we focused on analyzing the average coupling strength of the dominant delta/low gamma (fP: 0.5–4 Hz/fA: 30–70 Hz) and delta/high gamma (fP: 0.5–4 Hz/fA: 80–200 Hz) and found their coupling strengths were indifferent regardless of injury or strain (Figures 3B,C). Despite similar coupling strength, we observed a shift in the preferred phase of delta/gamma coupling from the rising phase of delta wave in CC031-sham control mice to the falling phase of delta wave in CC031-TBI mice who later developed PTE (Figures 3D,E).

**Figure 4:**
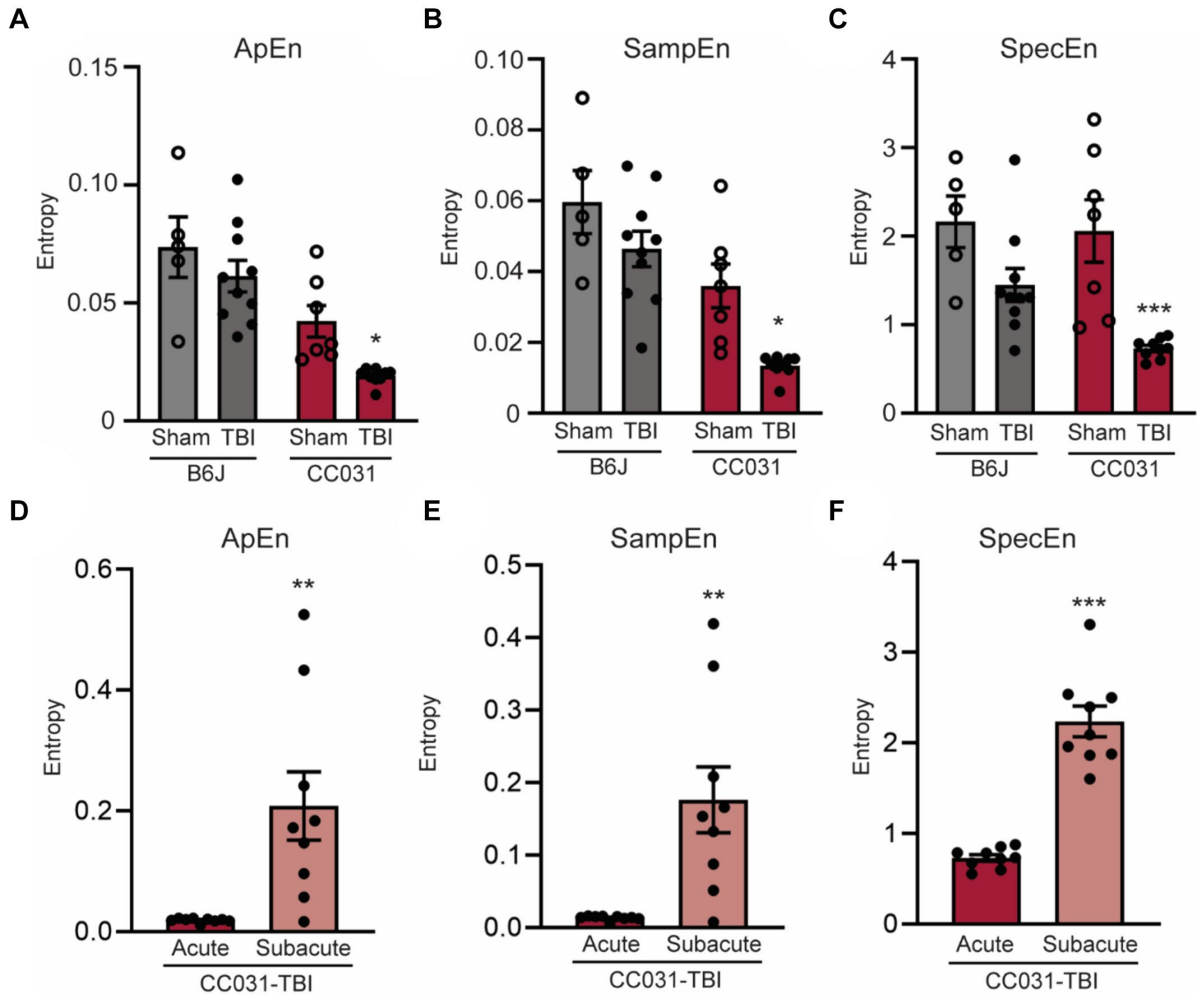
Lower early EEG entropy is dominant in the hippocampus of CC031 mice who develop PTE after TBI. Analyses of **(A)** Approximate entropy (ApEn), **(B)** sample entropy (SampEn), and **(C)** spectral entropy (SpecEn) in B6J-Sham, B6J-TBI, CC031-Sham, and CC031-TBI mice. Data are presented as mean ± SEM and analyzed using two-way ANOVA with Šídák’s multiple comparisons test, ^*^p < 0.05 and ^***^p < 0.001, *n* = 5–10. **(D)** ApEn, **(E)** SampEn, and **(F)** SpecEn of CC031-TBI mice were analyzed by comparing the acute vs. subacute phase. Data are presented as mean ± SEM and analyzed using paired Student’s *t*-test, *n* = 9, ^**^p < 0.01; ^***^p < 0.001.

Besides linear measurements, we also assessed the non-linear features like the randomness of LFP by computing approximate entropy (ApEn), sample entropy (SampEn), and spectral entropy (SpecEn). Our analysis revealed significantly lower randomness of LFP power and spectrum during the acute phase after TBI in CC031 mice who developed PTE compared to non-epileptic CC031-Sham, B6J-Sham, and B6J-TBI controls (Figure 4). Collectively, these quantitative early brain oscillatory alterations associated with PTE offer novel biological insights into how acute electrophysiological responses determine the risk of PTE and suggest potential targets for PTE prevention through neuromodulation.

## Discussion

Utilizing a novel mouse model of PTE within the CC panel, we conducted a systematic EEG biomarker study after TBI in a genetically diverse mouse population under controlled experimental conditions. Our findings reveal that DHRS is present exclusively during the acute phase in mice who developed PTE after TBI. The absence of such abnormalities in other mouse strains not exhibiting PTE after TBI suggests these oscillatory alterations could be associated with immediate pathological responses of the brain to trauma that drives epileptogenesis.

Clinically, our results hold significant implications on two fronts. Firstly, EEG is a crucial component of the multimodal recordings of the intensive care unit (ICU), where patients are admitted after sustaining a TBI (Claassen et al., 2013). Both acute and subacute EEG have been increasingly collected as a routine procedure after TBI to facilitate diagnosis and indicate the prognosis of TBI (Tewarie et al., 2023). Our findings align with a recent case–control study showing quantitative EEG features, including higher spectral power in the delta wave during early admission (within 3–5 days), can discriminate the risk of PTE after severe TBI (Pease et al., 2023). Secondly, EEG provides noninvasive real-time monitoring of brain states and offers non-linear measurement of the dynamic changes of oscillations, reflecting the biological functions of the brain. These properties position EEG as a promising biomarker for PTE.

Competing hypotheses have emerged about the potential cause of DHRS. The absence of DHRS from sham-operated CC031 mice or other non-epileptic mice strains excludes the possibility that the DHRS is a consequence of isoflurane anesthesia. Synchronized video recordings also confirmed that DHRS occurrences coincided with periods of rest in CC031-TBI mice, thus eliminating the possibility that immediate post-traumatic seizures or movement artifacts like self-grooming, eating, and drinking cause DHRS. However, we do not exclude the possibility that DHRS may reflect an episode of self-resolved non-convulsive status epilepticus (NCSE), which commonly occurs after TBI in both patients (Vespa et al., 1999) and animal models (Andrade et al., 2018). However, there are disputing diagnostic criteria for clinical NCSE, and there is a lack of a clear definition of preclinical electrographic NCSE (Zafar and Aljaafari, 2023). DHRS resembles some of the features of NCSE, including incrementing onset and duration >30 min, but their spiking frequency does not suffice the key criteria of NCSE of rhythmic discharges or spikes >0.5 Hz (Supplementary Figures 4, 5).

Identifying early DHRS that precedes PTE also sheds light on the potential mechanism of epileptogenesis after TBI. We speculate that DHRS may stem from a train of paroxysmal depolarization shifts in a large population of hippocampal CA1 pyramidal cells, which can be evoked by GABAa receptor inhibition, excessive glutamate release, or perturbed calcium homeostasis (Hotka and Kubista, 2019). Besides the power spectral features, our analyses of the phase-amplitude coupling and randomness of DHRS reveal immediate changes in the dynamic integration of neural activity following brain injury. For example, it is possible that primary phase preference could reflect the switch of hippocampal dynamic functional configuration between predominantly feedforward and feedback (Mariscal et al., 2021) during early epileptogenesis after TBI. We also speculate that the reduced entropy that leads to PTE could be a consequence of alterations in GABA transmission and, thereby, neuronal spike rate (Liang et al., 2015) after TBI. These multi-faceted quantitative EEG measures highlight the importance of considering multiple aspects of brain oscillations in understanding the pathophysiology of PTE.

Though DHRS offers a reliable biomarker in predicting PTE after FPI in CC mice, it is imperative to validate the generalizability of our findings by studying acute brain oscillation in other animal models of PTE. Notably, DHRS was also observed in one CC031 mouse but not in the rest of the CC or B6J strains that did not exhibit PTS during the same recording windows after TBI. However, longer continuous recordings are required to prove the absence of seizure in these mice, which is critical to determine whether the DHRS is PTE or strain genetic background dependent phenomena. While the injuries were induced during the light cycle and most DHRS were observed during the subsequent dark cycle, the light/dark and circadian effects on these brain oscillatory biomarkers can be further investigated under reversed light/dark or constant light cycles.

We collected LFP from a single bipolar electrode implanted into the contralateral hippocampus CA1 of mice. This recording paradigm not only allows us to capture generalized post-traumatic seizures that spread to the contralateral limbic system but also inform hippocampal oscillatory activities, which are critical to cognitive impairments that are commonly comorbid with PTE. More recording sites, particularly from bilateral cortical areas, are required to better understand the spatial distribution of DHRS, its relationship to potential epileptogenic zones, and the propagation of PTS. This is particularly important for translational implication with the notion that scalp EEG would be highly unlikely to detect hippocampal DHRS if it is not generalized to the superficial cortical areas. We also acknowledge that the inclusion of female mice is a critical next step in studying the sex effect on the brain oscillatory biomarkers of PTE. Further investigation using neuromodulatory approaches like phase-locked stimulation to suppress DHRS is required to ascertain a direct link between DHRS and PTE (Berényi et al., 2012). Despite these limitations, our study unveils serial early EEG characteristics preceding PTE, offering the potential for developing new therapeutic modalities and the prospect of ‘individualizing’ patient management based on their EEG findings. Though outside the scope of the current study, the genetic predisposition that renders CC031 more susceptible to DHRS and PTE can be further determined using genome-wide haplotype or targeted F2 quantitative trait locus mapping approaches that have been successfully implemented using CC (Noll et al., 2019).

## Data availability statement

The raw data supporting the conclusions of this article will be made available by the authors, without undue reservation.

## Ethics statement

The animal study was approved by the Institutional Animal Care and Use Committee of Ohio State University. The study was conducted in accordance with the local legislation and institutional requirements.

## Author contributions

TS: Investigation, Project administration, Writing – original draft, Writing – review & editing. NL: Data curation, Formal analysis, Investigation, Methodology, Visualization, Writing – original draft. RD: Data curation, Formal analysis, Visualization, Writing – original draft. YS: Data curation, Formal analysis, Visualization, Writing – original draft. CC: Investigation, Project administration, Writing – original draft. NR: Data curation, Writing – original draft. JF: Writing – review & editing, Methodology, Formal analysis. FP-M: Project administration, Resources, Writing – original draft, Writing – review & editing. OK-C: Conceptualization, Funding acquisition, Project administration, Resources, Supervision, Writing – original draft, Writing – review & editing. BG: Conceptualization, Data curation, Formal analysis, Funding acquisition, Investigation, Methodology, Project administration, Resources, Software, Supervision, Validation, Visualization, Writing – original draft, Writing – review & editing.
