# Supplementary material for: Early hippocampal high-amplitude rhythmic spikes predict post-traumatic epilepsy in mice
Source: Front Neurosci. 2024 Aug 29;18:1422449. doi: 10.3389/fnins.2024.1422449 (PMC11390562; doi:10.3389/fnins.2024.1422449)
Supplement: Supplementary file 1 [file Table_1.DOCX]

**Supplemental Materials**

**Supplemental figures**


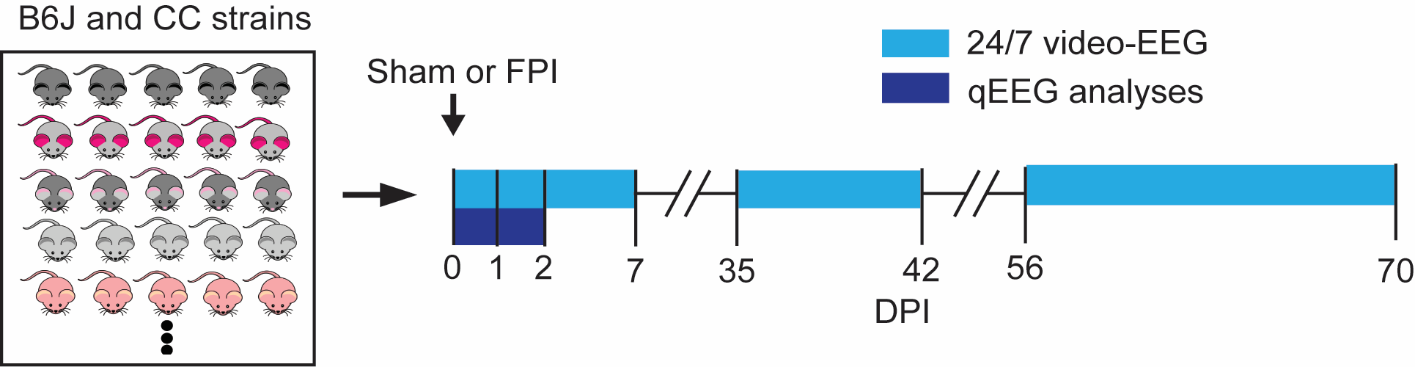


**Supplemental Figure 1. Schematic experimental design and recordings.**


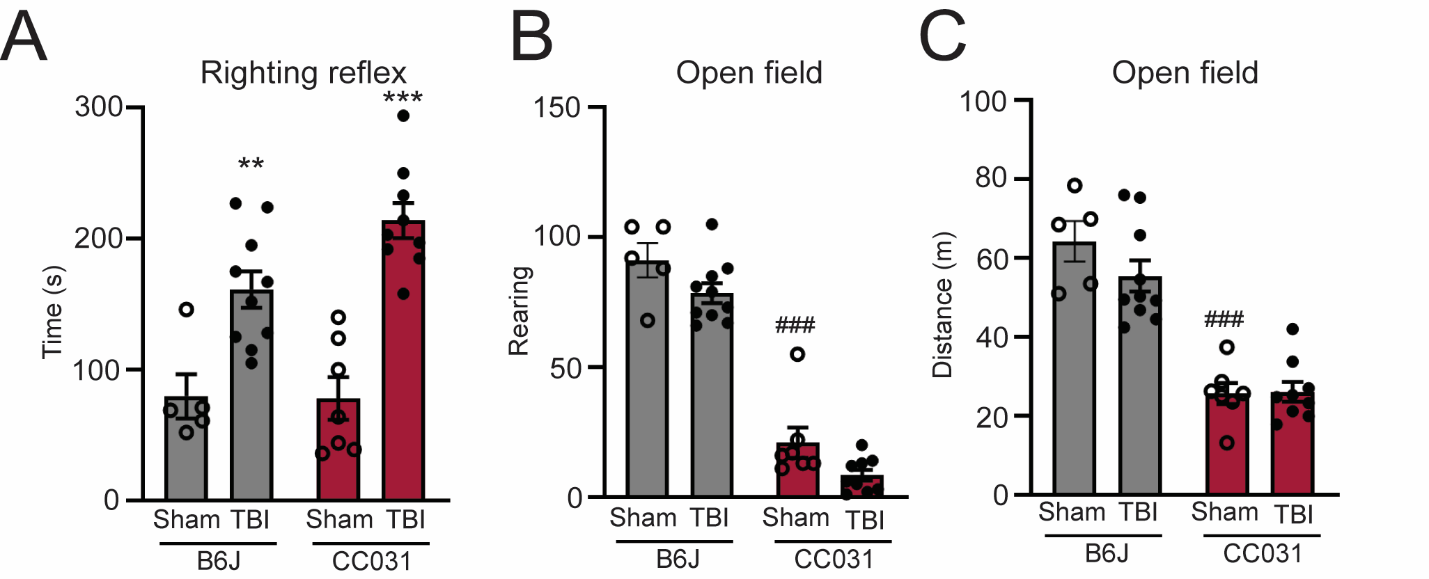
**Supplemental Figure 2. Neurological and locomotor measures of TBI in B6J and CC031 mice.** (A) Righting reflex time increased in B6J and CC031 mice immediately following TBI compared to sham operation. (B) The number of rearing and (C) distance traveled in an open field test (10 DPI) in B6J and CC031 mice experiencing sham or TBI. CC031 mice are more hypoactive compared to B6J regardless of injury. Data are presented as mean ± SEM and analyzed using two-way ANOVA with Šídák's multiple comparisons test. n = 5 – 10, **P < 0.01 compared to B6J-sham and ***P < 0.001 compared to CC031-sham; ^###^P< 0.001 compared to B6J-sham.


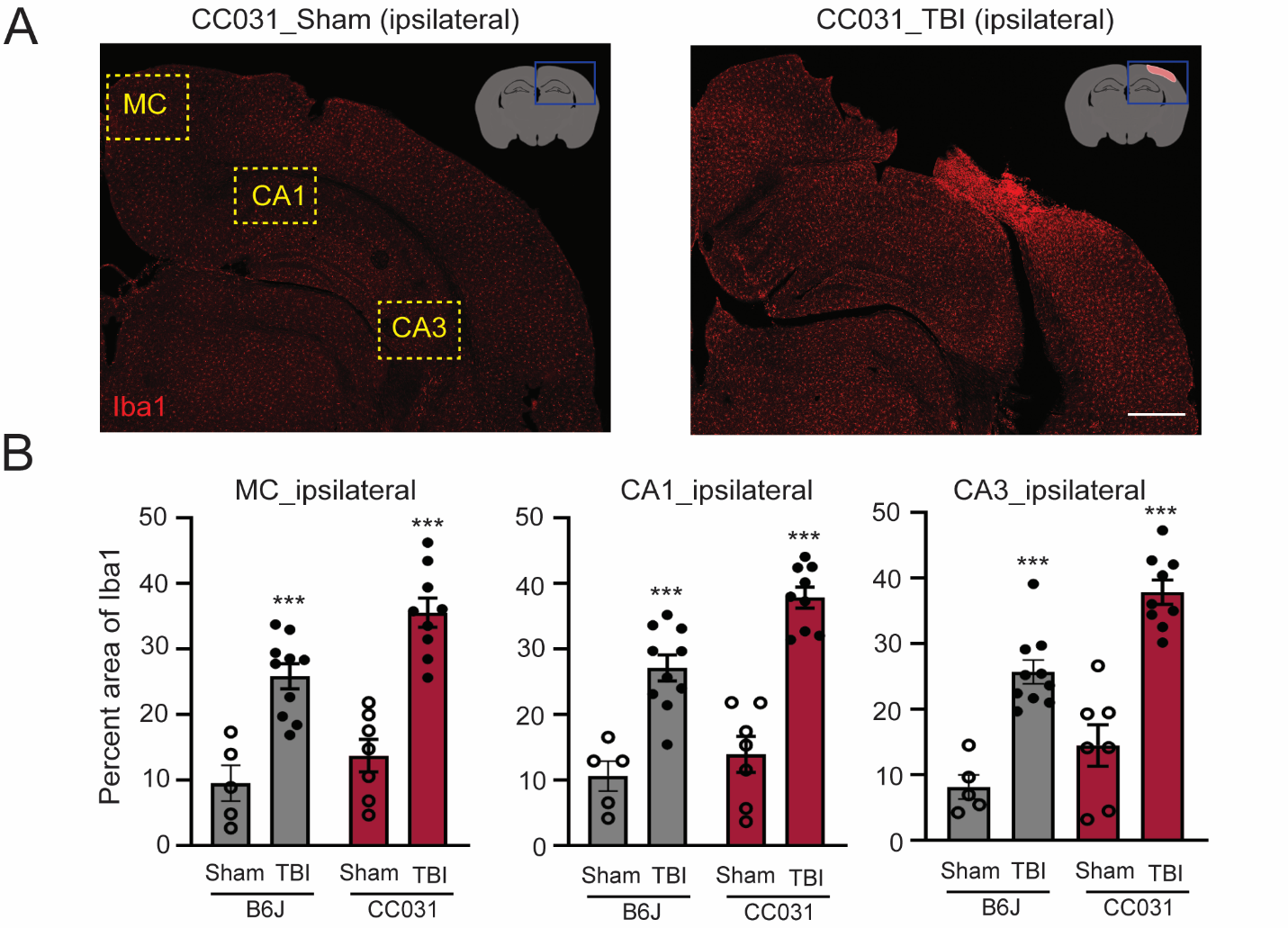
**Supplemental Figure 3. Iba1 immunointensity in B6J and CC031 mice in the presence or absence of injury.** (A) Representative images of Iba1 immunostaining of ipsilateral hemisphere in CC031-sham and CC031-TBI mice. Yellow dashed rectangles denote the medial cortex (MC), hippocampal CA1, and CA3. Scale bar: 500 μm. Inlets illustrate the outline of the region of interest relevant to the injury site. (B) Quantification of the percent area of Iba1 immunoreactivity above the threshold in ipsilateral MC, hippocampal CA1, and hippocampal CA3. Data are presented as mean ± SEM and analyzed using two-way ANOVA with Šídák's multiple comparisons test. n = 5 – 10, ***P < 0.001 compared sham controls.


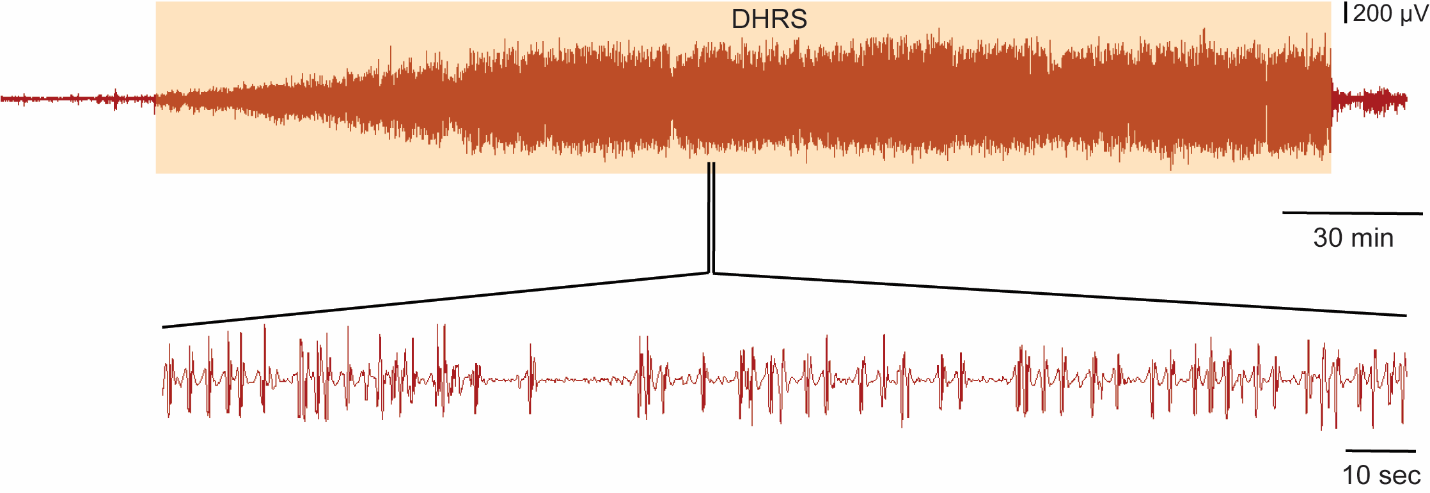


**Supplemental Figure 4. Representative hippocampal LFP traces showing the entire episode of DHRS and spikes in a CC031-TBI mouse.** This representative episode of DHRS features incrementing onset with temporal evolution that lasts ~4.2 hr.


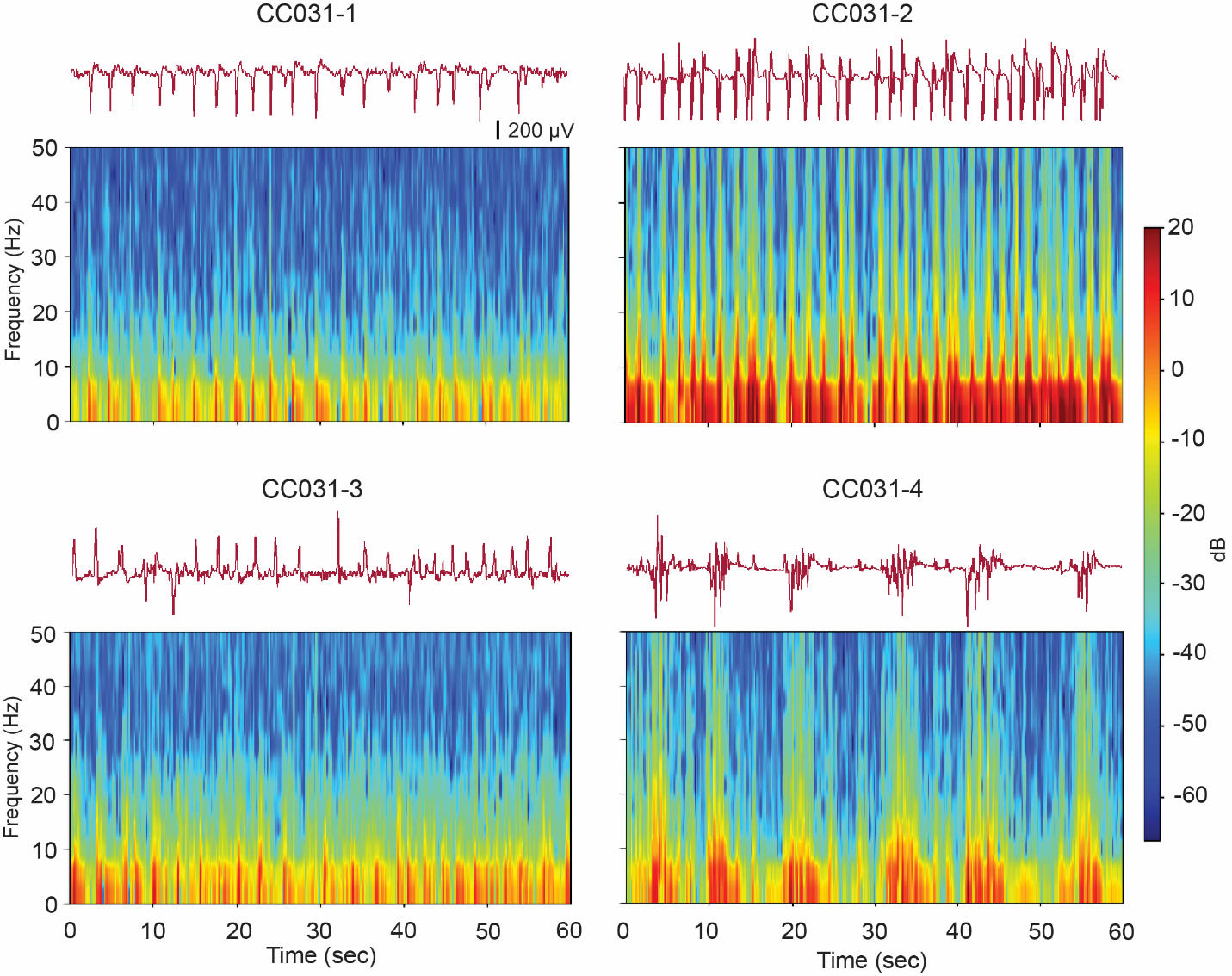


**Supplemental Figure 5. Representative hippocampal LFP traces and spectrograms of acute DHRS in CC031-TBI mice.** DHRS in CC031-1, CC031-2, and CC031-3 mice are characterized by 0.3–0.5 Hz high voltage rhythmic spikes. CC031-4 showed a unique DHRS pattern with bouts of 5 s spindles at a ~10 s interval.
